# Feasibility and Engagement of a Peer-Driven Mobile Intervention for Adolescent E-Cigarette Cessation: Cluster Randomized Pilot Study

**DOI:** 10.2196/79667

**Published:** 2026-03-17

**Authors:** Rajani Shankar Sadasivam, Elise M Stevens, Jessica Wijesundara, Bo Wang, Kavitha Balakrishnan, Reem Najjar, Christine Frisard, Narayani Ballambat, Lori Pbert

**Affiliations:** 1 Population and Quantitative Health Sciences Department, UMass Chan Medical School, 368 Plantation Street AS8-1081, Worcester, MA, 01605, United States, 1 (508) 856-8924

**Keywords:** e-cigarettes, vaping, adolescents, cessation strategies, mHealth, mobile health

## Abstract

**Background:**

E-cigarette use remains prevalent among US adolescents, with many reporting daily use and high nicotine dependence. Few evidence-based mobile health interventions focus specifically on adolescents.

**Objective:**

This study aimed to evaluate the feasibility, engagement, and preliminary efficacy of vaper-to-vaper (V2V)—a multicomponent, peer-driven texting intervention supporting adolescent e-cigarette cessation.

**Methods:**

A cluster randomized pilot study was conducted in 5 Massachusetts high schools, with schools randomized to either the V2V texting intervention (n=3) or a control group (n=2) that received a link to the National Cancer Institute’s Smokefree.gov Quit Vaping website. The V2V intervention included four components: (1) peer-written messages provided motivation, tips, and strategies to support adolescents in quitting vaping, sent daily in the first 30 days; (2) peer videos featuring adolescents sharing their experiences with e-cigarettes and motivations to quit, sent regularly as links aligned with related peer message topics; (3) peer coaches—university students aged younger than 22 years who had successfully quit vaping—trained to provide support, encouragement and answers to participants’ questions through the texting platform; and (4) a fictional, gamified mystery story integrated into the texting platform to promote engagement. Each gamified message included a short story segment and a question, with the next segment unlocked after a response or automatically after 3 days. The intervention was mainly delivered over 30 days, but adolescents could message the peer coach over the 3 months. Eligible participants (grades 9‐12, current e-cigarette users) were followed for 3 months. We assessed the feasibility of recruitment and retention (target: 80 participants, ≥85% retention), engagement with intervention components, and participant satisfaction. The secondary outcomes included improvements from baseline in confidence to quit, self-efficacy to resist vaping in specific high-risk situations, and fewer days vaped. E-cigarette cessation was biochemically verified using the Abbott iScreen cotinine test.

**Results:**

Seventy-one adolescents enrolled (intervention: 39/71, 55% ; control: 32/71, 45%), with a 96% follow-up rate at 3 months. Among intervention participants who responded to engagement items (N=37), high engagement—defined as self-reported use always, usually, or about half the time—was highest for peer messaging (n=29, 78%), followed by gamification (n=18, 49%), peer coaching (n=18, 49%), and peer video (n=13, 35%). The intervention group showed nonsignificant improvements in confidence to quit (n=17, 46%, vs n=9, 24%, moved from not at all, somewhat, or moderately confident to very or extremely confident) and in the number of days vaped in the past 30 days (−3.6 vs −2.9), while self-efficacy scores (adapted smoking self-efficacy scale range 12‐60) were slightly lower compared to the control group (mean −0.21, SD 1.14, vs mean 0.06, SD 1.39). Cotinine-validated 7-day point prevalence abstinence was similar between groups (intervention: 21.6% vs control: 22.6%).

**Conclusions:**

The V2V intervention demonstrated feasibility and acceptability, with strong engagement and high satisfaction. Although differences between groups were not statistically significant, findings suggest that peer-driven mobile interventions are a promising approach to support adolescent e-cigarette cessation.

## Introduction

On the basis of the 2024 National Youth Tobacco Survey, an estimated 2.25 million adolescents reported using e-cigarettes (also known as vapes) [1]. While these numbers indicate a decline from 2.8 million in 2023, e-cigarettes remain the most commonly used tobacco product among adolescents [1,2]. A significant portion of adolescents who continue to use e-cigarettes report daily use and high levels of addiction [3]. Most e-cigarettes contain nicotine, a highly addictive substance that adversely affects adolescent brain development, impacting learning, memory, attention, mood, and impulse control [4]. In a national study among middle and high school adolescents, e-cigarette dependence was more prevalent among youth who reported vaping in the past compared to those who did not (17.6% vs 5.2%; *P*<.001), with many reporting cravings, urges, a strong need to use, and difficulties with concentration and mood [3]. In line with this national study, adolescents participating in formative work reported higher levels of nicotine dependence and strong urges to vape from e-cigarettes and found quitting very challenging [5,6]. Effective interventions are needed to help adolescents quit e-cigarette use.

Despite this need, there are very few evidence-based cessation interventions that exist for adolescent e-cigarette users and even fewer that leverage the digital health modalities that adolescents already use daily. To our knowledge, *This is Quitting*—a text messaging intervention developed by the Truth Foundation—is the only mobile health (mHealth) program to date that has been empirically tested with adolescents in a randomized trial [7]. A recent trial comparing *This is Quitting* to an assessment-only control found that it significantly increased point prevalence abstinence rates at 7 months, demonstrating the potential of mHealth to promote e-cigarette cessation among adolescents [7]. We also found one app, Crush the Crave, that is currently being tested among adolescents [8], with other programs such as the ACT on Vaping mobile app and the Pivot program being evaluated with young adults and adults, respectively [9,10]. Adolescents vary in their readiness to quit and may have different preferences for engaging with behavioral interventions. Therefore, a range of digital intervention options is necessary to expand support for adolescents who are addicted to e-cigarettes.

Thus, we developed the vaper-to-vaper (V2V) intervention—a multicomponent, peer-driven texting intervention. Peer influence is a key driver motivating adolescents to vape [11]. Previous research with adolescents, including our own, has shown that peer-driven interventions can counteract negative social norms associated with quitting (eg, feeling left out when close peers still use e-cigarettes) and improve outcomes [12-16]. Additionally, our previous smoking cessation work with adolescents has demonstrated that they are highly receptive to peer coaching [16]. The V2V intervention builds on insights from our work developing peer-driven mHealth programs for adults who smoke but are not yet ready to quit, recognizing that adolescent e-cigarette users present similar engagement challenges [17,18]. These strategies included proactive pushed content through text messaging, peer videos, and incorporating gamified experiences [19].

In this paper, we described the V2V intervention and presented findings from a pilot randomized study. The primary goal of this pilot study was to assess the feasibility of the study design and protocol implementation, collect key process measures (such as engagement with the intervention), and generate preliminary estimates of effect sizes for health-related outcomes [20-22]. In alignment with these guidelines, our primary hypothesis assessed the feasibility of recruiting and retaining adolescents who vape into a school-based digital cessation program (target: 80 participants, ≥85% retention). We also evaluated engagement with and satisfaction toward the V2V intervention, including interactions with text messages, peer coaching, and videos. Finally, we explored preliminary effects of V2V on vaping outcomes, hypothesizing that intervention participants would demonstrate higher confidence to quit, greater self-efficacy to resist vaping in specific high-risk situations, fewer days vaped, and higher cotinine-validated abstinence compared to controls.

## Methods

### Study Design

Our pilot feasibility cluster randomized study was conducted in 5 high schools in Massachusetts. Three schools were randomized to the V2V intervention, and 2 schools were randomized to the control. Adolescents assigned to the control arm were emailed a link to the National Cancer Institute’s Smokefree.Gov Quit Vaping website [23]. We followed participants for 3 months and assessed feasibility and cessation outcomes. The study was conducted during the COVID-19 pandemic, which impacted school operations and led to adjustments in both the development timeline and recruitment period. Participants were recruited between November 2022 and March 2023.

### Ethical Considerations

The study was approved by the UMass Chan Medical School Institutional Review Board (IRB H00021082). All participants participated in the study voluntarily. For adolescents aged younger than 18 years, parental assent was used in lieu of the informed consent (informed assent), whereas informed consent was obtained from participants older than 18 years before their enrollment in the study. Informed assent, or informed consent if the student is aged 18 years or older, was explained such that students understood they had a right to refuse to participate, that their care in the school health clinic and their grades would not be affected by whether or not they decided to participate, and that confidentiality would be maintained. Participants’ identifiable information was deidentified before data analysis to ensure their privacy and confidentiality. A Data Safety Monitoring Board monitored the progress of the study and met with the study team twice a year. Participants received US $25 for completing the baseline and another US $25 for completing the 3-month follow-up.

### Participants and Inclusion and Exclusion Criteria

To recruit adolescents, we mailed study information letters and a fact sheet to all parents (or legal guardians) of grade 9 to 12 students in participating schools, informing them of the study and asking them to contact us or the school within 2 weeks of receiving the letter if they did not want their child to participate in the study. Although the intervention was delivered in English, study materials were provided in the five most common languages across participating schools—English, Spanish, Brazilian Portuguese, Arabic, and Vietnamese—to support recruitment of adolescents from households where other languages were primarily spoken. After 2 weeks, the study was announced in health classes, school-wide announcements were made, and posters were placed throughout the school. All recruitment materials included the study’s research coordinator’s contact information, and students were asked to contact the study’s research coordinator if they wished to participate. Nurses also encouraged students to talk with the research coordinator. We also noted in our posters and study materials that we did not share information about the participants with the schools unless the participant indicated suicidal ideation or intention to do harm. The research coordinator visited these schools once a week to enroll students in the study and to conduct the screening and baseline interviews in a private room.

Inclusion criteria included (1) enrollment in grades 9 to 12 at a participating high school, (2) currently e-cigarette user, (3) ownership of a smartphone, (4) English speaking, and (5) not having opted out. Current e-cigarette use was defined as at least 1 or more days to the question: “during the past 90 days, on how many days did you use e-cigarettes?” This eligibility criterion was adapted from the 2018 National Youth Tobacco Survey, a survey of US middle and high school students that used a 30-day time frame. Our study excluded those who were unable or unwilling to provide informed assent or consent (in the case of those aged ≥18 y).

### Intervention and Comparison Arms

#### Intervention Arm

Our team developed the V2V intervention components with the help of a panel of adolescents (N=11) from a high school in Massachusetts, different from those participating in our pilot study. The panel participants provided informed consent if aged older than 18 years; for those aged younger than 18 years who were not opted out by their parents, assent was obtained. Our study with this panel, conducted in 2022, has been published [5,6]. Among panel participants, 27% (n=3) identified as female, 36% (n=4) identified as male, 27% identified as nonbinary (n=3), and 10% preferred not to say (n=1). The majority of the panel was (n=8, 72%) in 10th or 11th grade [5,6]. All participants vaped nicotine, and half of the participants reported not thinking of quitting. Panel participants received a US $20 gift card as compensation for their participation in the development of each of the 4 intervention components for a possible total of US $80.

In the following sections, we described the development process of each V2V intervention component and how they were delivered within the intervention.

#### Component 1: Peer Messaging (Prewritten, Tailored, and Push Messages via Texting)

##### Development Process

These peer messages were created using a procedure we have successfully used in our previous studies [24]. We asked the panel participants to respond to scenarios created based on literature reviews and the results of our qualitative study, as reported in our prior papers [5,6]. The scenarios described common challenges faced by adolescents at various phases of their e-cigarette cessation journey—whether they were not yet ready to quit, setting a quit day, amid a quit attempt, or in maintenance. Additionally, we incorporated scenarios addressing interactions with family and friends, as this was identified as a common challenge for adolescents addicted to e-cigarettes. The instructions directed the adolescents to write the messages as if they were sending a quick text, using language, abbreviations, emojis, and so on, that they would typically use when texting a friend. Each team member rated each of the messages written by the adolescents independently, followed by a group review of the top-rated messages for relevance and appropriateness. From this process, our core team selected the set of 54 messages.

##### V2V Intervention Delivered

Once participants from the 3 intervention high schools were enrolled in the V2V intervention, they received daily peer messages during the initial 30 days. Message frequency was reduced to sending twice or thrice weekly for the subsequent 2 months. Participants could choose the time of day they received these messages (7 AM, 11 AM, or 6 PM).

### Component 2: Peer Videos (8‐10 Videos): 6 Adolescents and 18 Videos

#### Development Process

Peer videos included videos of peers describing their experiences using e-cigarettes, motivations to quit, and efforts to quit using them. To create these videos, we recruited members of the panel (n=6) to describe their experiences with vaping and their efforts to quit vaping in the videos. We created a script to guide these responses based on various phases of their e-cigarette cessation journey. To be eligible, the adolescents had to express a willingness to be videotaped. We requested active parental consent as well as adolescent assent for adolescents aged younger than 18 years before videotaping the peer videos. Additionally, we required all adolescents aged younger than 18 years and their parents to sign a video release authorization form before filming the peer videos. Those aged older than 18 years had to provide written informed consent and sign the video release authorization. Adolescents were compensated for the completion of this activity with a US $30 gift card.

#### V2V Intervention Delivered

To share peer videos, we paired them with peer messages covering similar topics. These links were sent at regular intervals to intervention participants over the first 30 days of the intervention.

### Component 3: Peer Coaching (Asynchronous Communications With Trained Coaches via Texting)

#### Development Process

Peer coaches—university students aged younger than 22 years who had successfully quit vaping (with difficulty)—were hired for their lived experience and interest in behavior change counseling. Before engaging with study participants, the coaches completed a comprehensive 2-month training that included human subjects research CITI (Collaborative Institutional Training Initiative) certification, study protocol review, instruction on using the texting platform, and guidance on tailoring communication with templated messages. They also received educational resources about the hazards of youth vaping and completed online motivational interviewing modules focused on adolescent substance use. In addition, peer coaches participated in role-playing sessions with the study’s MPI (multiple principal investigator) and a Certified Health Education Specialist specializing in substance use and tobacco cessation. These sessions, along with case study discussions on adolescent e-cigarette cessation, equipped coaches with the skills to handle a wide range of vaping-related questions and scenarios. Finally, the study team developed a structured peer coaching protocol that detailed procedures and expectations, including participant enrollment, communication timelines, and texting platform use. The protocol emphasized timely, empathetic, and personalized communication grounded in motivational interviewing techniques and included guidance for addressing mental health concerns.

#### V2V Intervention Delivered

The peer coaches provided support, encouragement, and answers to questions that study participants had about vaping and quitting. All communications and interactions occurred asynchronously over text messages through the study’s texting program. The peer coach received a notification when a participant proactively texted them and was expected to review and reply to vaping-related text messages from study participants within 24 to 48 hours of receiving the texts. Coaches also received notifications when participants were sent specific motivational texts, prompting them to reach out with templated texts to build rapport. The peer coaches had access to templated text message responses that could be tailored to specific questions about vaping and quitting. The peer coaches also met weekly with the study team to review motivational interviewing techniques and to get advice on handling complex texts and challenging situations.

### Component 4: Gamification (2 Game Narratives)

#### Development Process

To enhance intervention engagement, we incorporated a fictional, interactive mystery story based on advice from our panel. The goal was to use the game to encourage active participation. To design this game, we consulted our panel, asking them to identify their preferred top one or two games and to consider how they would develop a narrative for these games if given the opportunity. Their insights helped shape the storyline, which we then refined with a game developer.

#### V2V Intervention Delivered

Participants received text messages, each containing a snippet of the story, followed by a gamification question each day. The next portion of the story was only sent after the previous one was responded to or automatically resumed after 3 days.

### Brief Instructions to the Participant

Once participants completed consent and baseline procedures, our staff also provided brief instructions on each component of the intervention using a script developed by the team. Participants were informed that they would receive text messages written by their peers and the approximate time frame during which these messages would be delivered. Our staff also told the participants that some messages would include links to short peer videos and encouraged them to click on the links to view them. In addition, participants were informed that a peer coach would reach out to them and that they could also initiate contact with the coach at any time. Finally, participants were told that they would receive a detective-themed interactive story that they could engage with and solve over the course of the program. Our staff asked those assigned to the intervention group to save the phone numbers from which the study messages would be delivered. They then sent a test text message to the participants to make sure it went through. Furthermore, the staff asked the participant what time they wanted to receive the peer messages (7 AM, 11 AM, or 6 PM) and informed them that the detective story messages were going to be sent around 7 PM.

### Comparison Arm

At baseline, both control and intervention participants were provided a link to the National Cancer Institute SmokeFree Quit Vaping website [23]. The website included resources such as reasons to quit vaping, building a quit plan, strategies to quit vaping, and tips for dealing with cravings.

### Measures and Data Collection

Baseline and 3-month follow-up data were collected from all participants in the privacy of the school nurse’s office and on a study-issued tablet provided by the study staff, which included a link to the REDCap (Research Electronic Data Capture) survey. Data were collected by the research team to reduce the fear of repercussions for participating in the study. Baseline data included demographics and e-cigarette and other tobacco use. Additionally, the baseline survey assessed psychosocial factors, including perceived stress, anxiety, and depressive symptoms. Engagement was measured via self-report using a series of items beginning with the prompt, “How often did you…” followed by references to specific intervention components. Responses were recorded on a 5-point Likert scale, ranging from “always” to “never.” Satisfaction was an adapted version of the Client Satisfaction Questionnaire scale [25]. Secondary outcomes assessed at both baseline and follow-up included confidence to quit vaping, self-efficacy to resist vaping in specific high-risk situations, and the number of days vaped during the past 30 days. Confidence to quit was assessed with a single-item measure of perceived ability to quit vaping. Self-efficacy to resist vaping was assessed using a 12-item scale adapted from the Smoking Self-Efficacy Questionnaire [26]. The primary outcome measure was the 7-day point prevalence vaping cessation, assessed using the question: “Do you currently vape (vaped even 1 puff in the last 7 days)?” Biochemical verification was conducted for those who self-reported as quitters using the Abbott iScreen Cotinine Test for qualitative detection of cotinine in oral fluids at a 30 ng/mL cutoff level. This test detects up to 1 to 4 days after nicotine use. Participants were asked to actively swab the inside of their mouth and tongue with a sponge swab and then insert the collector vertically into the cap and twist the handle to tighten the cap on the test tube. Results (positive or negative) were read and displayed on the device’s screen after 10 minutes. A positive result indicated the presence of cotinine above the cutoff level, suggesting recent nicotine use. A negative result indicated cotinine levels below the cutoff, suggesting no recent nicotine use. Gift cards worth US $25 were provided to adolescents for completion of each assessment (baseline and 3-mo; US $50 total).

### Randomization

We randomized at the school level to minimize within-school contamination. To reduce the chances of imbalance across conditions [27], the schools were matched based on the percentage of low income, percentage of African American students, and number of students. They were then grouped into 2 matched pairs. Within each pair, 1 school was randomly assigned to each study condition. A fifth school was added to the study arm with the smaller total number of students to maintain balance in the overall sample size.

### Power and Sample Size Calculations

As pilot studies do not provide meaningful estimates of effect sizes due to the imprecision inherent in small samples [20,28], we based our proposed sample size of 40 students per condition (20 per school) on obtaining precision in estimating condition-specific retention rates, providing important information for the design and recruitment for the future R01 study. On the basis of our prior study of adolescent smoking cessation, the estimated intraschool correlation of 0.01 [29]. This corresponds to a design effect due to within-school clustering of 1+[0.01×(20–1)]=1.19, resulting in an effective sample size per condition of 40/1.19=34. Assuming 85% retention, the final analytic sample size per condition is expected to be approximately 29 participants. Thus, a conservatively wide 95% exact CI for the per-condition retention rate is (68.9%-95.0%).

### Data Analysis

Group comparisons for demographic characteristics and tobacco-related behaviors at baseline are presented as the N and proportion or mean (SD) in each group with a *P* value from a chi-square, Fisher exact, or *t* test, as appropriate. As the number of other tobacco products used in the past 30 days was highly skewed, a Wilcoxon rank sum test was used for the *P* value. Engagement and satisfaction items are presented as the N and proportion for categorical variables and mean or median, SD, and range for continuous variables. Change in confidence to quit was measured by dichotomizing the response options and measuring whether someone moved from not at all, somewhat, or moderately confident to very or extremely confident (+1), vice versa (−1) or stayed in the same category (0). Results are reported as N and proportion with a *P* value from a chi-square test. Self-efficacy to resist vaping was calculated as the sum of 12 items (5-point Likert scale, ranging from “not at all” to “extremely”; range 12‐60) asking for the adolescent’s perceived ability to avoid vaping in a variety of different emotional or social situations and was presented as the mean (SE). Change in past 30-day use of e-cigarettes was presented as the mean (SE) and was obtained from a linear regression model. Self-reported quitting and cotinine-validated quitting at 3-month follow-up were analyzed using a logistic regression model among adolescents with completed follow-up (completed cases). Models for vaping cessation outcomes were further adjusted for baseline confidence to quit and the number of other tobacco products used. Owing to the small number of schools and the small sample size, we used model fit statistics to compare models both with and without a random effect for school or an adjustment for school. A random effect for school was not included in the models as the amount of variance due to the random effect was 0 and models were not adjusted for school as school is confounded with randomization assignment. We used the past 90-day inclusion criterion to be maximally inclusive, recognizing that many adolescents vape intermittently rather than daily or weekly. This broader window allowed us to capture a wider range of vaping patterns, including occasional users who may still experience nicotine dependence but might not report past 30-day use. As including these users could potentially dilute intervention effects, we conducted sensitivity analyses for our primary and secondary outcomes excluding participants who reported no vaping in the past 30 days. Additionally, as use of other tobacco products at baseline could impact the vaping cessation outcome, we also conducted a sensitivity analysis excluding all those who reported using any other form of tobacco. Analyses were conducted in Stata/MP version 19.0 (Stata Corp) for Windows (Rev. October 6, 2025).

## Results

### Overview

**Figure 1.**
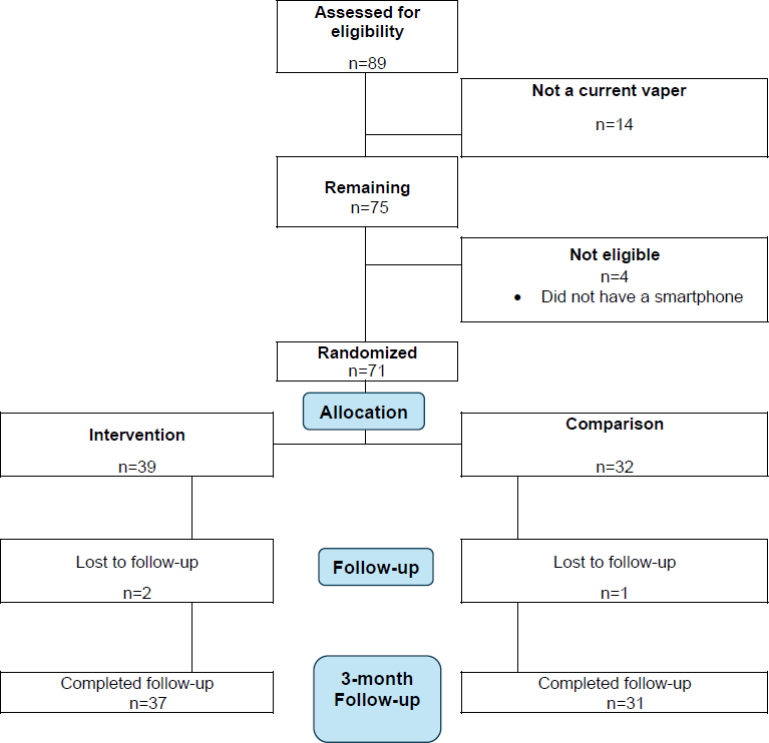
CONSORT (Consolidated Standards of Reporting Trials) flow diagram of a cluster randomized pilot study evaluating the feasibility and preliminary outcomes of the vaper-to-vaper mobile peer-driven texting intervention for adolescent e-cigarette cessation in 5 Massachusetts high schools, 2022 to 2023.

Overall, we recruited 71 participants for the study (control group: n=32, 45%, and intervention group: n=39, 55%). We achieved a follow-up rate of 95.8% (Figure 1).

Table 1 describes the demographic characteristics. The intervention group included a higher proportion of non-Hispanic White participants versus the control (n=28, 73.7%, vs n=15, 46.9%) and a lower proportion of Hispanic participants (n=5, 13.2%, vs n=15, 46.9%; *P*=.008). Additionally, more students in the intervention group were in higher grade levels—11th and 12th grades combined (n=27, 69.3%, vs n=12, 37.5%)—compared to the control group (*P*=.004). The control group had lower self-efficacy than the intervention group at baseline (*P*=.05).

**Table 1.** Baseline characteristics of adolescents who vape (N=71) enrolled in a cluster randomized pilot study of the vaper-to-vaper mobile text messaging intervention versus control, conducted in 5 Massachusetts high schools, 2022 to 2023.

Characteristics	Randomization assignment		*P* value
	Control (n=32)	Intervention (n=39)	
Age^a^, n (%)			.24
15	2 (6.3)	3 (7.7)	
16	12 (37.5)	6 (15.4)	
17	10 (31.3)	14 (35.9)	
18	6 (18.8)	9 (23.1)	
19	2 (6.3)	7 (17.9)	
Age, mean (SD)	16.8 (1.0)	17.3 (1.2)	.08
Gender^a^, n (%)			.94
Male	16 (50)	17 (43.6)	
Female	16 (50)	20 (51.3)	
Transgender	0 (0)	1 (2.6)	
Nonbinary	0 (0)	1 (2.6)	
Race or ethnicity, n (%)			.008
Non-Hispanic White	15 (46.9)	28 (73.7)	
Non-Hispanic non-White	2 (6.3)	5 (13.2)	
Hispanic	15 (46.9)	5 (13.2)	
What grade are you in^a^?, n (%)			.004
9	3 (9.4)	6 (15.4)	
10	17 (53.1)	6 (15.4)	
11	8 (25)	12 (30.8)	
12	4 (12.5)	15 (38.5)	
Retained at 3-month follow-up	32 (100)	36 (92.3)	.37
Perceived Stress Scale (range=0-16; higher score=higher perceived stress), mean (SD)	7.4 (3.1)	7. 5 (3.5)	.91
Depressive symptoms (range=16-34; higher score=more depressive symptoms), mean (SD)	27.3 (4.5)	27.7 (4.6)	.69
Confidence to quit—If you were to decide to quit vaping, how confident are you that you could stop completely, n (%)			.37
Not at all	3 (9.7)	2 (5.3)	
Somewhat	6 (19.4)	11 (29.0)	
Moderately	6 (19.4)	13 (34.2)	
Very	7 (22.6)	6 (15.8)	
Extremely	9 (29.0)	6 (15.8)	
Self-Efficacy Questionnaire (range=1-5; higher score=higher self-efficacy to restrain from vaping), mean (SD)	2.4 (.81)	2.7 (.72)	.05
Hooked on Nicotine Checklist (range=0-10; higher score=more addicted), mean (SD)	5.0 (3.6)	5.2 (3.4)	.78

a *P* values were calculated using chi-square tests when all expected cell counts were >5 and Fisher’s exact tests when any expected cell count was <5

Participants in both groups reported depressive symptom scores in the higher range of the scale, suggesting elevated depressive symptom burden (intervention: mean 27.7, SD 4.6; and control: mean 27.3, SD 4.5). Most participants in both groups reported cannabis use in the past 30 days (intervention: n=23, 59%; and control: n=18, 56.2%; Table 2).

**Table 2.** Tobacco-related behaviors and “other” substance use at baseline among adolescents (N=71) who vape enrolled in a cluster randomized pilot study of the vaper-to-vaper mobile text messaging intervention versus control, conducted in 5 Massachusetts high schools, 2022 to 2023.

	Control (n=32)	Intervention (n=39)	*P* value
Number of days vaped in the last 30 days, mean (SD)	17.8 (12.2)	19.7 (11.0)	.48
Past 30 days other tobacco use, n (%)			
Chewing tobacco, snuff, or dip	0 (0)	4 (10.3)	.12
Cigarettes	1 (3.1)	6 (15.4)	.12
Blunts, cigars, and cigarillos	6 (18.8)	9 (23.1)	.77
Hookah	2 (6.2)	5 (12.8)	.45
Tobacco in a pipe (including water pipe)	0 (0)	1 (2.6)	>.99
Past 30 days marijuana use, n (%)	18 (56.2)	23 (59)	>.99
Past 30 days vaped THC (tetrahydrocannabinol), n (%)	13 (45)	20 (53)	.62

### Engagement (Intervention Only)

**Figure 2.**
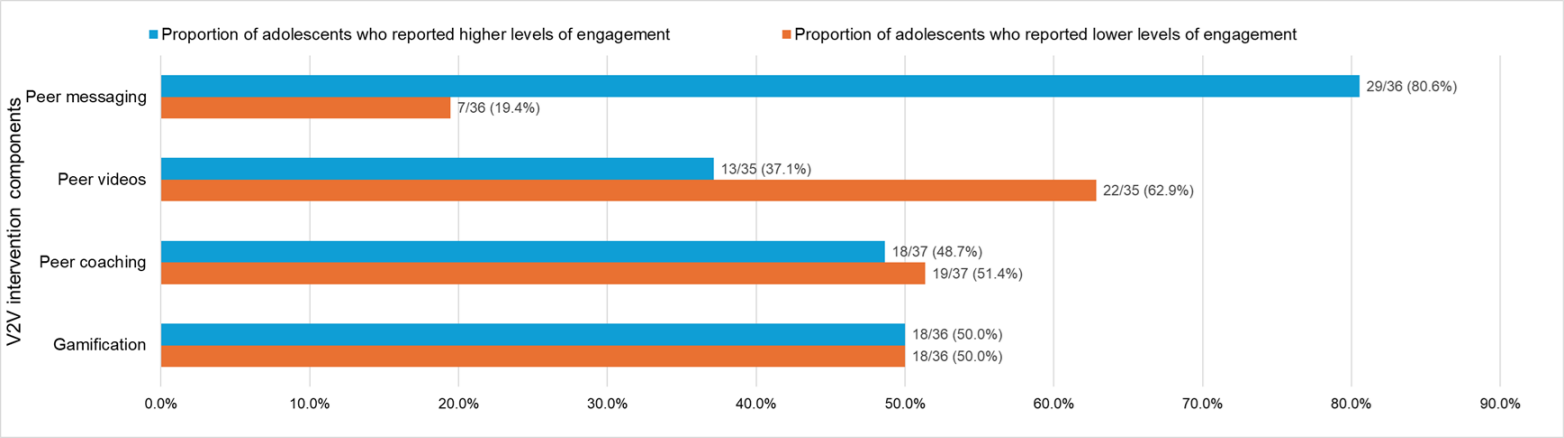
Engagement with intervention components among adolescents randomized to the vaper-to-vaper mobile texting intervention in a cluster randomized pilot study conducted in Massachusetts high schools, with outcomes assessed over the 3-month follow-up period (n=39). Participants who selected “Prefer not to answer” were excluded from percentage calculations. Higher engaged included those who responded with "Always,” “Usually,” or “About half the time," and lower engaged included those who responded with "Never,” or “Rarely."

More than 80% of participants (n=29) reported being highly engaged with peer messaging, whereas the percentages of higher engagement with gamification and peer coaching were 50% (n=18) and 49% (n=18), respectively. Engagement with peer videos was lower, with only 37% of participants (n=13) reporting high engagement (Figure 2). The Engagement Summary Score, ranging from 0 to 4, had a mean of 2.1 (SD 1.4).

### Satisfaction (Intervention Only)

The Satisfaction Summary Score for the intervention group had a mean of 25.3 (SD 4.7). The summary score is the sum of 8 items, ranging from 1 to 4 (range 8‐32), and higher scores indicate higher satisfaction.

### Secondary Outcomes

#### Confidence to Quit Vaping

In the control group, of the 50% of participants who reported a change in confidence at 3-month follow-up, 28% (n=9) reported higher confidence, and 22% (n=7) reported lower confidence. Of the 74% of intervention participants reporting a change, 44% (n=17) reported higher confidence, and 31% reported lower confidence (n=12; Table 3). In our analysis, limited to participants who reported vaping in the past 30 days at baseline, 33% (n=8) reported higher confidence, and 21% (n=5) reported lower confidence. Among these intervention participants, 43% (n=14) reported higher confidence, and 30% (n=10) reported lower confidence.

**Table 3.** Secondary outcome: changes in confidence to quit, self-efficacy to resist vaping, and number of vaped in the last 30 days among adolescents (N=71) participating in a cluster randomized pilot study of a peer-driven mobile texting intervention (vaper-to-vaper) versus control in Massachusetts high schools.

Category	Control (n=32)	Intervention (n=39)	*P* value
Confidence to quit, n (%)			.11
Poorer	7 (21.9)	12 (30.8)	
Same	16 (50)	10 (25.6)	
Better	9 (28.1)	17 (43.6)	
Change in, mean (SD)			
Self-efficacy to resist vape in different situations	0.06 (1.39)	−0.21 (1.4)	.39
Number of days vaped in the last 30 d	−2.9 (10.0)	−3.6 (10.8)	.79

#### Self-Efficacy to Resist Vaping

The mean changes in self-efficacy scores (SEQ-12) from baseline to follow-up did not differ significantly between groups (control: mean 0.06, SD 1.39; and intervention: mean –0.21, SD 1.14; *P*=.39; Table 3). The results were similar after limiting to participants who reported vaping in the past 30 days at baseline (control: mean 0.04, SD 1.01; and intervention: mean –0.36, SD 1.13; *P*=.16).

#### Number of Days Vaped in the Last 30 Days

Both groups reported a decrease in the number of days they vaped in the previous 30 days (control: mean −2.9, SD 10.0, vs intervention: mean −3.6, SD 10.8). None of these findings were statistically significant (Table 3). The sensitivity analysis limited to participants who reported vaping in the past 30 days at baseline did not change our results (control: mean −2.3, SD 10.1, vs intervention: mean −3.3, SD 11.2).

#### Vaping Abstinence

At the 3-month follow-up, cotinine-validated 7-day point prevalence was not statistically significant (intervention: n=8, 21.6%, vs control: n=7, 22.6; *P*=.90). Using an unadjusted logistic regression model, the intervention group had a nonsignificant 5% reduction in the odds of cotinine-validated quitting compared to the control group (odds ratio [OR] 0.95, 95% CI 0.30‐2.99), which changed to a 30% increase in the odds of cotinine-validated quitting after adjustment for confidence to quit at baseline and the number of other tobacco substances used (OR 1.30, 95% CI 0.35‐4.89; Table 4). In a sensitivity analysis limited to participants who reported vaping in the past 30 days at baseline, the intervention effect remained nonsignificant (unadjusted OR 1.06, 95% CI 0.30‐3.80; and adjusted OR 1.18, 95% CI 0.29‐4.90), indicating that excluding noncurrent users did not meaningfully change the results. The results did not alter meaningfully (OR 0.83, 95% CI 0.18-3.88) after excluding all those who reported using other tobacco products at baseline.

**Table 4.** Changes in vaping abstinence outcomes from baseline to 3-mo follow-up by treatment condition among adolescents with completed follow-up (n=68) participating in a cluster randomized pilot study of a peer-driven mobile texting intervention (vaper-to-vaper) versus control in Massachusetts high schools.

Variable	Control (n=32), n (%)	Intervention (n=39), n (%)	*P* value	Unadjusted OR^a^ (95% CI)	Adjusted for confidence to quit at baseline and number of other substances
Self-reported vaping abstinence	9 (29.0)	12 (32.4)	.76	1.17 (0.42‐3.31)	2.18 (0.58‐8.11)
Cotinine-verified vaping abstinence	7 (22.6)	8 (21.6)	.92	0.95 (0.30‐2.99)	1.30 (0.35‐4.89)

a OR: odds ratio.

As a number of participants reported cannabis use and this can impact the outcome, we also conducted a stratified analysis comparing those who used cannabis at baseline and those who did not. Regardless of intervention condition, those who reported using cannabis at baseline had significantly lower odds of cotinine-validated quitting than those who did not report using cannabis at baseline (OR 0.20, SE 0.13; *P*=.01). Results for self-reported quitting were consistent with cotinine-validated quitting (OR 0.25, SE 0.14; *P*=.02). Overall, 22% of those who reported using cannabis at baseline reported not using at follow-up (n=9; *P*<.001). There were no differences by randomization assignment.

## Discussion

### Principal Findings

We were able to recruit 71 participants (with a target of 80), despite COVID-19–related school closures and delays. While our recruitment window was shortened (from 5 mo to 2.5), enrollment progressed efficiently once we were able to initiate the study. Importantly, we achieved an excellent 96% follow-up rate, demonstrating strong participant retention and study feasibility. Engagement with the intervention varied between the components. Participants in the intervention group also reported higher acceptance and satisfaction with the components compared to the control. The intervention group showed improvements in confidence to quit and the number of vapes per day in the last 30 days, although self-efficacy to resist vaping slightly reduced, with actual quitting rates not significantly differing between groups.

Our findings add to the growing evidence that mobile and text-based interventions are feasible and acceptable tools for engaging adolescents in health behavior change [30-35]. Adolescents have near-universal mobile phone ownership and frequently use text messaging [36]. Text messaging and mobile programs have been used successfully to support youth tobacco cessation by enhancing motivation and maintaining engagement [31]. As previously reported, the evaluation of *This is Quitting* demonstrated that text messaging can also be adapted to support vaping cessation [7]. Vaping cessation apps (Crush the Crave) are currently undergoing testing among adolescents [8]. To enhance accessibility and real-world applicability, we implemented our text messaging program in a school-based setting. This approach was adapted from our previous work on recruiting and engaging adults who are not yet ready to quit, with the assumption that adolescents may have similar problems with engagement and retention [17,18]. Our previous approach included text-based and peer-driven components—peer-written messages and videos, asynchronous peer coaching, and gamified storytelling—each designed to increase engagement and relevance [19]. By integrating these elements within a school-based program, we aim to both maximize participation and retention and real-world accessibility of our program for adolescent participants.

The adolescent participants noted high levels of acceptance and satisfaction with the intervention, which is important to consider for future iterations of the intervention. The peer-written messages were well-received by our participants. Consistent with prior studies, interventions that push content (such as text messages) tend to be more engaging and effective than content that a participant has to choose to view (like content on a website) [35,37,38]. Text from a peer may increase homophily—a feeling of similarity between the message writer and the message reader—which in turn can increase relevance and engagement with the provided content [24]. Prior studies have demonstrated that peer-written messages can effectively promote health behaviors among youth. Adolescents in one study wrote text messages that emphasized care, responsibility, and personal relevance to encourage friends to seek HIV or STI (sexually transmitted infection) testing, indicating the impact of text messaging among the youth [39]. Observational research also suggests that peer-to-peer communication is frequent among adolescents and strongly influences social and emotional outcomes [36]. Peer-to-peer communication, with a focus on text messaging, serves as an effective way to disseminate health information, promoting healthier behaviors, especially with the use of addictive substances such as e-cigarettes.

Peer coaching may be a promising approach to engage and help adolescents who are addicted to vaping. In a qualitative study of adolescents and young adults, peer coaching integrated into social media–based cessation groups was viewed favorably, with participants emphasizing the importance of age similarity, shared lived experience, and mentor training [40]. Such coaching can be delivered synchronously (eg, through live chat or video sessions) or asynchronously, as in our study. Evidence from prior work suggests that asynchronous peer coaching can be both practical and cost-effective, although its engagement and effectiveness vary across contexts [41-43]. This format may be particularly well-suited for school-based and youth interventions, as it can be private and allows adolescents to engage in their own time. In our study, most of the adolescent participants engaged at least once with the peer coach, although the frequency of engagement varied (range 0‐17). One lesson may be that the peer coach needs to take a more proactive role—initiating contact rather than waiting for participants to reach out. Similar proactive outreach models by peers have been shown to promote engagement in studies that tested peer communication in a social media or discussion forum setting [44]. Alternate or adaptive content strategies—such as personalized follow-up messages—could also enhance engagement. Future studies should further test these approaches to identify the best ways to promote engagement with and the effectiveness of the intervention among adolescents.

Engagement with the gamification messages was evenly split. Rather than using strategies such as points or rewards [45], we chose to use a fictional story informed by our formative work to promote engagement. We assumed that if adolescents found the storyline engaging, it could provide a brief respite from the challenging task of quitting vaping and encourage greater participation in other intervention components. Furthermore, in our formative work, participants expressed interest in having multiple storylines, and future studies providing more choices may spur more engagement [5,6]. However, due to time and budget constraints, we only had one gamification story in our intervention—a detective story—which may not have been of interest to some participants. Including additional storylines to offer participants varied options may further enhance engagement with the gamification messages. Peer videos were the component with the lowest engagement. There may have been several factors. First, the way we distributed these peer videos (via links on text messages) may have been a barrier for adolescents. Second, the quality of the video recording was perhaps lower than what adolescents are used to when they visit social media sites such as TikTok or Instagram. Future studies should consider using these channels on social media to distribute these videos to further increase engagement with peer videos.

Of concern, at baseline, many of our participants reported elevated depressive symptom scores on the depressive symptom scale, as indicated by a mean score of 27. These levels may have been higher due to the study being conducted during the COVID-19 pandemic. Several studies, including 2 meta-analyses, showed an increase in depression symptoms during the COVID-19 pandemic [46,47]. We did not find studies that estimated the levels of depression among adolescents after the COVID-19 pandemic. Depression can lead to poorer outcomes among tobacco users, and future vaping cessation studies may need to assess and include methods to address depression among their participants.

Furthermore, our participants also reported high levels of cannabis use and THC vaping [48-50], reflecting broader trends of e-cigarette and cannabis co-use over the last few years. Notably, co-use of cannabis and vaping THC can also reduce the effectiveness of tobacco interventions [49,51], although the degree to which this co-use affects cessation has not been fully studied. This was evident in our study: in a secondary analysis, those who did report cannabis use at baseline had significantly lower odds of cotinine-validated quitting (OR 0.20, SE 0.13; *P*=.01), compared to those who did not report cannabis use at baseline. An interesting future research study might be to compare vaping cessation interventions that address co-use versus those that do not [52]. This might yield valuable data on how to treat adolescents who both vape and co-use cannabis.

Among the secondary outcomes, our results indicated that the intervention produced mixed changes in participants’ confidence levels, with some reporting decreases and others increases in confidence. The intervention included content related to understanding addiction and the challenges of quitting, as well as practical strategies to quit. This may have helped some participants gain a better understanding of the difficulties involved in quitting, while others found the strategies useful for strengthening their confidence to quit. A similar pattern was observed in changes in self-efficacy, where control participants had a slightly greater increase in scores than intervention participants. While intervention participants may have gained new knowledge about coping and quitting strategies, greater awareness of the challenges associated with resisting vaping may have reduced their self-efficacy scores. A longer duration of exposure to the intervention may help overcome this initial reduction effect, although this needs to be tested in future studies. Intervention participants reported a greater reduction in the number of days vaped in the past 30 days compared to controls, suggesting some potential benefit of the intervention, although the absolute difference was small.

The difference in quitting rates between the control and intervention groups was not statistically significant. However, our inclusion criterion (vaping in the past 90 days) may have been broad and allowed participation of adolescents who had already quit or substantially reduced vaping. As few participants reported not vaping in the past 30 days, we conducted sensitivity analyses excluding these participants. The findings from these analyses remained consistent with the main results, suggesting that the inclusion of occasional or former users did not meaningfully alter the observed intervention effects. Overall, our results indicate some promising effects of the intervention but also highlight areas for refinement to further enhance its potential impact in future trials.

Our study had several limitations. The sample size of our pilot study was small, limiting the ability to examine differences in intervention effects between participants with higher versus lower rates of e-cigarette use. We also used e-cigarette use within the past 90 days for inclusion into the study, instead of the 30-day window. This may have resulted in the recruitment of less frequent users or those who had already quit. As noted, we added sensitivity analyses to minimize this potential bias. Engagement was assessed through self-report only. In addition, satisfaction was reported only among participants in the intervention group, which limited our ability to compare satisfaction outcomes between study groups. As several participants reported use of other tobacco products, cotinine testing may not have distinguished between those who quit vaping but continued using other products; to reduce the impact of this limitation, we report sensitivity analysis results of the cotinine-validated vaping cessation outcome excluding all these participants. Additionally, the intervention was implemented over a short period due to the challenges posed by the COVID-19 pandemic, which resulted in our development time being reduced from the planned 1 year to 4 months. This may have constrained its impact, given the challenges of quitting e-cigarettes; a longer intervention period might yield stronger effects.

### Conclusion

We demonstrated the feasibility of recruiting and randomizing participants from high schools in Massachusetts, achieving high rates of follow-up. The study revealed several promising aspects of the intervention that warrant further investigation. These include the overall acceptability and satisfaction scores, as well as higher levels of engagement with certain components, such as peer messaging and peer coaching. Although vaping cessation rates did not increase compared to the control group, intervention participants demonstrated mixed but encouraging changes in confidence, greater reduction in the number of days vaped, and slightly lower self-efficacy to resist vaping compared to the control group. These findings support the feasibility and potential of the V2V intervention and provide direction for optimizing the intervention and testing it in a larger, adequately powered trial.

## Supplementary material

10.2196/79667 Checklist 1 CONSORT-eHEALTH checklist (V 1.6.1)

## Abbreviations

mHealth: mobile health
OR: odds ratio
V2V: vaper-to-vaper
